# Impact of High-Intensity Interval Training (HIIT) on Patient Recovery After Myocardial Infarction and Stroke: A Fast Track to Fitness

**DOI:** 10.7759/cureus.73910

**Published:** 2024-11-18

**Authors:** Abhishek Kumar, Manisha Gupta, Abhijeet K Kohat, Arpit Agrawal, Amratansh Varshney, Ankit Chugh, Deepak I Koshy, Ramesh Gurjar, Pritish Kumar

**Affiliations:** 1 Cardiology, All India Institute of Medical Sciences, Raipur, Raipur, IND; 2 Neurology, DKSPGI (Dau Kalyan Singh Postgraduate Institute) Raipur, Raipur, IND; 3 Internal Medicine, All India Institute of Medical Sciences, Raipur, Raipur, IND; 4 Internal Medicine, Pandit Jawahar Lal Nehru Memorial Medical College, Raipur, IND

**Keywords:** cardiovascular rehabilitation, exercise training, high intensity, interval, myocardial infarction, stroke

## Abstract

Regular exercise leads to various adaptations and many pathophysiological changes that significantly benefit exercise stamina and overall health, both in the general population and in patients with chronic cardiovascular diseases. High-intensity interval training (HIIT) is a form of exercise training that consists of short repetitive bursts of intense workouts that push the body to more than 90 percent of its maximal oxygen consumption (VO2 max) and more than 75 percent of maximal power, with in between periods of low-intensity exercise for recovery, followed by a cool-down period. HIIT has unquestionably emerged as a powerful and effective intervention in rehabilitating patients, particularly those recovering from heart attacks and strokes. HIIT significantly enhances cardiovascular fitness by improving parameters such as VO2 max, endothelial function, and overall cardiac output. In addition to these cardiovascular benefits, HIIT also contributes to improved metabolic health, including better glycemic control and lipid profile regulation, which are often compromised in patients with cardiovascular conditions. Furthermore, HIIT has a positive impact on stroke patient's functional recovery and quality of life, allowing them to regain mobility, independence, and a sense of well-being more quickly. While concerns about the safety of high-intensity exercises in individuals with compromised heart function remain, current evidence suggests that when properly supervised, HIIT is both safe and well-tolerated in these populations. As healthcare continues to evolve, the incorporation of innovative and evidence-based approaches such as HIIT may redefine the future of cardiovascular rehabilitation, ultimately providing long-term health benefits for patients recovering from MI and stroke.

## Introduction and background

Myocardial infarction (MI) and stroke are very common cardiovascular diseases (CVDs) and are among the foremost causes of death and disability worldwide, accounting for roughly 31% of all fatalities globally according to The Global Burden of Cardiovascular Diseases and Risk 2022 by Vaduganathan et al. [1]. Cardiovascular rehabilitation (CVR) is crucial for secondary prevention of CVDs. It aims to improve rehabilitation outcomes, reduce the risk of further cardiac events, alleviate psychological distress like anxiety and depression, and enhance overall quality of life (QOL). CVR includes educational programs about heart health, lifestyle behavior modifications, psychosocial support, and supervised exercise regimens. Engaging in physical workouts or outdoor activity is as important as a non-pharmacological approach to managing CVDs. Regular physical activity is more effective than many other treatments in enhancing health-related QOL, as it fosters beneficial cardiovascular changes and stimulates the release of mood-enhancing neurotransmitters. Despite the well-documented benefits of exercise, approximately 1.4 billion people globally, including one-third of the adult population and more than 80% of adolescents, do not meet current recommendations of health guidelines for exercise as per their age [1]. This makes physical inactivity a global problem.

While moderate-intensity continuous training (MICT) has primarily been highlighted in conventional rehabilitation strategies, high-intensity interval training (HIIT) has recently gained recognition as a valuable alternative. HIIT involves short bursts of vigorous exercise performed at a relatively high workload. This corresponds to at least 90% of maximal oxygen consumption (VO2 max), over 75% of maximal power, at least 90% of the maximal target heart rate, and a perceived exertion level of 6 or higher on a 10-point Borg scale, or 15 or higher on a 6-20 Borg scale. HIIT has demonstrated significant advantages in improving cardiovascular fitness, metabolic health, and recovery for individuals recovering from MI and stroke. It results in more significant physiological adaptations than MICT, which typically entails 30 to 60 minutes of exercise at only 40% to 60% of the oxygen consumption reserve as seen in the study by MacInnis et al. [2]. Despite the established effectiveness of CVR in enhancing functional capacity and overall health-related QOL, access to these programs remains limited, especially in lower and middle-class income countries. There is a pressing need for innovative home-based and technology-assisted CVR models to improve accessibility and meet the needs of an aging population facing multiple chronic conditions. Future studies should aim to broaden the evidence supporting CVR across diverse patient demographics and conditions, while also investigating sustainable delivery methods that can expand access to CVR services worldwide.

## Review

Pathophysiology

**Figure 1:**
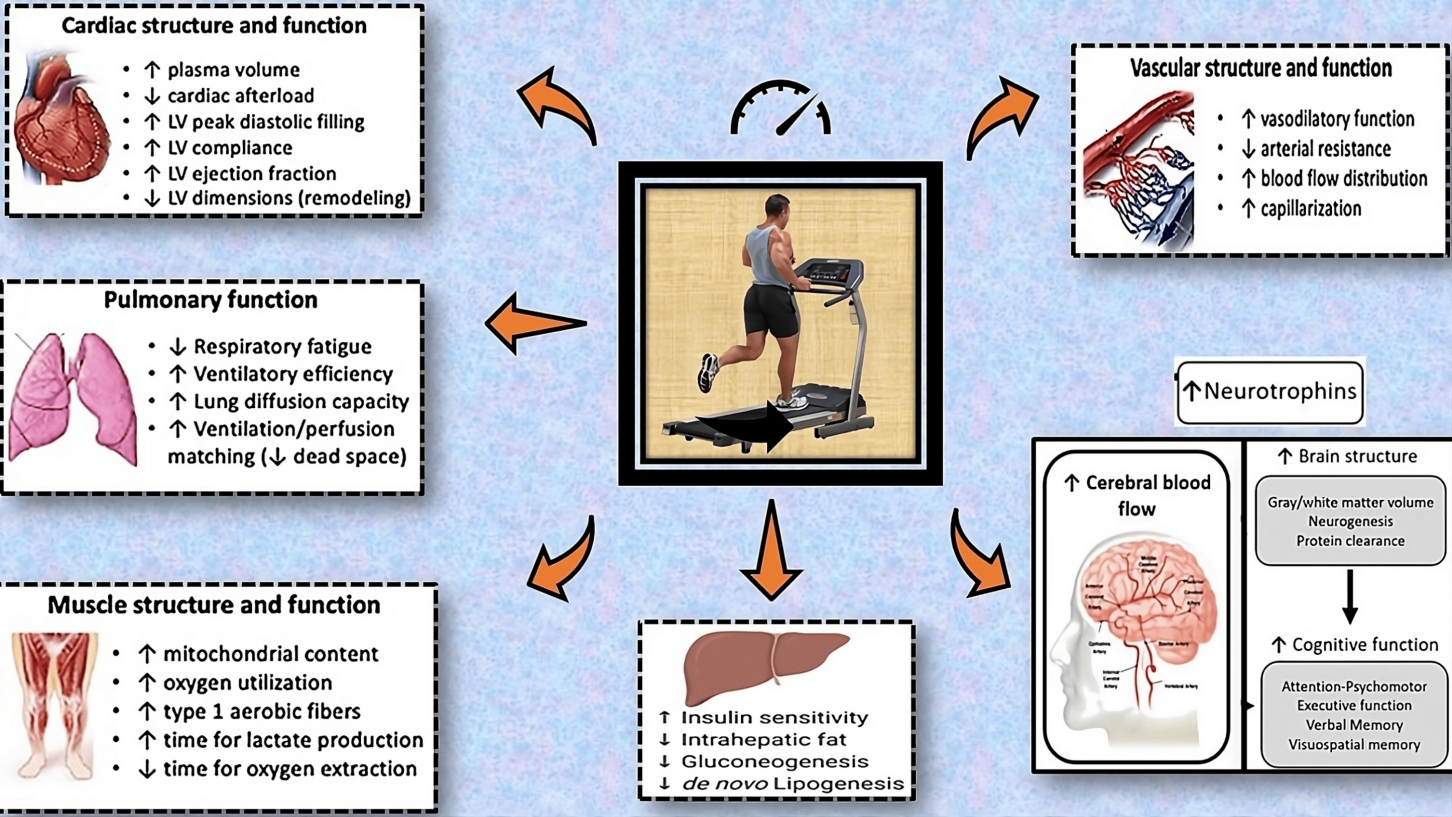
Overview of the pathophysiology and positive influence of high-intensity interval training (HIIT) on cardiac and neuro health. Image Credit: Authors

HIIT has been shown to induce several physiological adaptations that are particularly beneficial for patients recovering from MI and stroke. These adaptations include improvements in cardiac remodeling, autonomic regulation, metabolic flexibility, motor function, muscle strength, and balance (Figure 1).

Cardiovascular Adaptations of HIIT in Cardiac Rehabilitation

Increased maximal oxygen uptake (VO2 max): Oxygen utilization is a critical indicator of cardiovascular fitness and overall health. Weston et al. in their meta-analysis found that HIIT led to a 19.4% greater increase in VO2 peak among patients with cardiometabolic diseases compared to MICT (P < 0.05) [3]. The aerobic capacity and VO2 max improvement resulting from the exercise relies on various peripheral and central adaptations. These adaptations include enhancement of the central nervous system's capacity to recruit motor units, elevated stroke volume and cardiac output by the heart, and increased capillary density, oxygen extraction, and oxidative enzyme capacity by skeletal muscles. Oxygen oxygen-carrying capacity of the blood is also increased due to an increase in the number, volume, and hemoglobin content of red blood cells. This enhancement in VO2 max is particularly noteworthy as it is associated with improved aerobic capacity and a decreased risk of cardiovascular events.

Enhanced endothelial function: Endothelial dysfunction, a precursor to atherosclerosis and cardiovascular events, can be measured through flow-mediated dilation, which is regulated by nitric oxide (NO) availability. HIIT has been shown to enhance endothelial function by increasing NO availability and promoting vascular remodeling, probably because of the increased shear stress during the active workout phase. The heightened availability of NO in the endothelium contributes to enhanced flow-mediated dilation, resulting in increased perfusion and oxygen delivery to peripheral tissue. Additionally, it has been found to reduce mean blood pressure, with a more significant impact on diastolic blood pressure. Kleinloog et al. demonstrated that HIIT enhanced endothelial function by increasing flow-mediated dilation, by up to 2.23% in patients with type 2 diabetes after an eight-week intervention (P < 0.05) [4]. This improvement indicates enhanced vascular health and reduced cardiovascular risk.

Improved lipid profile: HIIT has been associated with favorable changes in lipid metabolism, by reducing low-density lipoprotein (LDL) levels and elevating high-density lipoprotein. The meta-analysis by Milanović et al. reported that HIIT led to significant reductions in LDL cholesterol levels, with an average decrease of 10 mg/dL compared to MICT (P < 0.05) [5]. Participants in the HIIT group also experienced an increase in high-density lipoprotein (HDL) cholesterol mean value by 3 mg/dL, further improving their lipid profile. Additionally, HIIT enhances metabolic flexibility, allowing the body to efficiently switch between carbohydrate and fat oxidation. Terada et al. in a study found that even a single session of HIIT reduces the area under the curve for same-day postprandial glucose in individuals with pre-diabetes and type 2 diabetes and is linked to a reduction in the duration of time during which glucose levels are ≥ 10 mmol/L [6]. These adaptations also lead to better insulin sensitivity by increasing muscle oxidative capacity and glucose transport capacity, resulting in better control of blood sugar levels, as well as beneficial cardiovascular changes. They also documented a rise in the peroxisome proliferator-activated receptor-gamma coactivator 1 alpha (PGC-1α), which is known as the principal regulator of mitochondrial biogenesis in muscle, following HIIT [6]. Elevated PGC-1α levels have a positive impact on skeletal muscle’s oxidative capacity, antioxidant defense, and resistance to age-related muscle loss.

Reduced inflammation: Chronic inflammation is a major factor in cardiovascular diseases. HIIT has been shown to decrease levels of interleukin-6 (IL-6) and C-reactive protein (CRP), important inflammatory markers, indicating a decrease in systemic inflammation with regular HIIT sessions. HIIT training may also increase free radical production, activate the NF-KB signaling pathway, and boost heat shock proteins. HIIT can improve circulating adiponectin, leptin, and TNF-α, which may help control low-grade inflammation in people with metabolic disorders. A recent meta-analysis done by Tuttle et al. has shown that there is a significant inverse relationship between skeletal muscle mass and inflammatory markers [7]. Furthermore, it has been suggested that the cytokines released from muscles during exercise have direct anti-inflammatory effects and play a role in improving cardiometabolic health.

Cardiac remodeling: HIIT promotes favorable cardiac remodeling by enhancing left ventricular function and reducing the heart failure risk. Exercise stimulates rapamycin mTOR signal transduction pathway which leads to the upregulation of ribosomal biogenesis and protein synthesis. This in turn induces physiological hypertrophy. A study by Huang et al. demonstrated that HIIT improved left ventricular ejection fraction (LVEF) and stroke volume in patients with chronic heart failure, indicating its potential to aid recovery post-myocardial infarction [8]. Naseer et al. have shown that 3-D LVEF and speckled tracking echocardiographic strain parameters are the latest and most significant echocardiographic parameters associated with poor cardiovascular outcomes [9]. In another pilot study involving post-acute myocardial infarction patients done by Manresa et al., HIIT led to significant improvements in echocardiographic parameters related to left ventricular function after 12 weeks of training (P < 0.05) [10]. In persons with metabolic syndrome, increased cardiac torsion reflects endocardial fiber damage. HIIT leads to reduced cardiac torsion and early filling pressure of the left ventricle in adults with myocardial infarction and type 2 diabetes indicating a potential decrease in endocardial damage.

Autonomic regulation: HIIT positively affects autonomic regulation by improving heart rate variability (HRV) and hence is associated with better cardiovascular outcomes and reduced mortality risk in cardiac patients. Manresa et al. in their study found that HIIT significantly improved HRV in coronary artery disease (CAD) patients, suggesting a beneficial impact on autonomic function [10]. This benefit of improved cardiac autonomic control is seen in both healthy individuals as well as in patients with CAD. The effects of HRV are due to an increase in parasympathetic intonation because of the increased availability of nitric oxide, which stimulates vagal neurons. This is reflected in the linear indexes of rMSSD (the square root of the mean differences between consecutive high-squared RR intervals), pNN50 (the percentage of successive differences between normal adjacent intervals greater than 50 ms), and a decrease in the ratio between low and high frequencies. Additionally, HIIT increases carotid artery distensibility, which in turn leads to improvements in baroreflex sensitivity.

Neurological Adaptations of HIIT in Stroke Rehabilitation

Neuroplasticity: HIIT enhances neuroplasticity by promoting the formation of new neural connections. This is especially important in stroke recovery, as it may help compensate for damaged areas of the brain by utilizing healthy adjacent regions. A study by Mang et al. revealed that HIIT enhanced motor learning in healthy individuals, indicating its potential to promote neuroplasticity in stroke survivors [11]. Boyne et al. have studied several potential factors that facilitate the neuroplasticity processes, memory, and cognitive improvements after doing HIIT [12]. During exercise, skeletal muscles release lactate. Elevated blood lactate levels are often associated with higher serum brain-derived neurotrophic factor (BDNF) levels, increased motor cortex excitability, and improved motor learning. The upregulation of BDNF expression in the hippocampus and cortex, along with its high affinity for receptor tropomyosin receptor kinase B, promotes neuron survival, neurogenesis, and synaptic plasticity. These are often linked to improvements in memory. Furthermore, it has been observed that genes associated with gamma-aminobutyric acid (GABA) are downregulated during exercise. GABA is an inhibitory neurotransmitter and a decrease in GABA function increases BDNF levels, which in turn facilitates neurogenesis during exercise. Boyne et al. also found that even a single session of HIIT can increase blood lactate concentrations, BDNF as well as vascular endothelium-derived insulin-like growth factor 1 levels without any significant stress cortisol response [12]. Cortisol on the other hand is known to suppress neuroplasticity developments. These findings also correlated similarly with four-week HIIT as compared to MICT.

Cerebral blood flow: HIIT can increase cerebral blood flow, which is essential for delivering oxygen and nutrients to the brain. Improved cerebral perfusion may enhance recovery of motor function and cognitive abilities in stroke patients. The main mechanism behind this is improved endothelial function and better autonomic regulation as described earlier. Tsukamoto et al. reported that HIIT increased cerebral blood flow in healthy adults, which could translate into similar benefits for stroke survivors by enhancing brain recovery mechanisms [13]. They also found higher deoxyhemoglobin concentrations in the ipsilesional hemisphere of ischemic stroke patients doing HIIT as compared to MICT, suggesting better cerebral and systemic oxygen consumption.

Increased muscle strength: HIIT can lead to significant improvements in muscle strength, which is crucial for mobility and independence in stroke survivors. The HIIT Stroke Study found that stroke patients engaging in HIIT exhibited greater improvements in muscle strength compared to those participating in traditional rehabilitation programs [14]. MacInnis et al. found that HIIT induces and increases the contents and activities of mitochondrial biomarkers such as citrate synthase and oxidative phosphorylation complexes in human skeletal muscle compared to MICT [2]. Additionally, they observed a significant increase of more than 50% in calcium reuptake, which improved the work capacity of the muscles and contributed to fitness improvements. Hence, they concluded that due to different patterns and intensities of muscle contraction, HIIT induces greater mitochondrial response compared to MICT.

Enhanced motor function, balance, and coordination: It is shown that HIIT improves motor function and coordination in stroke patients. The repetitive nature of high-intensity exercise can help retrain the brain and improve neuromuscular connections. Gjellesvik et al. in HIIT Stroke Study have demonstrated significant improvements in walking distance and balance among stroke survivors participating in HIIT combined with standard care compared to those receiving standard care alone [14]. Specifically, the intervention group showed a 28.3-meter improvement on a six-minute walk test (P = 0.030) and a 1.27 points improvement on the Berg Balance Scale (P = 0.025) immediately after the HIIT intervention. High-intensity exercises often require quick changes in direction and body positioning, which can enhance balance and coordination in stroke patients. Improved balance reduces the risk of falls, a common concern in this population.

Clinical evidence

To understand the link between HIIT and cardiovascular disease outcomes, a comprehensive literature search was conducted from database inception through 2005 utilizing various electronic databases such as PubMed, Cochrane Library, EMBASE, Scopus, and the International Clinical Trials Registry (ICTR) Platform. The search employed keyword terms: cardiovascular disease, myocardial infarction; ‘cardiovascular rehabilitation/rehabilitations’; ‘cerebrovascular disease, stroke’; ‘exercises/exercise, high-intensity, intermittent, interval, training’; ‘moderate-intensity continuous training; and their related terms. The inclusion criteria were: (1) randomized controlled trials (RCTs) associating the effectiveness of HIIT with MICT; (2) patients with CAD and stroke as study population; (3) measurement of at least three of the following outcomes: VO2 peak, HR peak, blood pressure, six-minute walk test (6MWT), LVEF, Berg Balance Scale (BBS), neural plasticity markers, Dynamic Gait Index, cognition; and (4) the use of Cox regression or multivariable logistic regression for statistical adjustment. Narrative reviews, case reports, studies only in abstracts or conference proceedings, and non-peer-reviewed studies were excluded. Numerous studies have provided evidence supporting the efficacy of HIIT in improving outcomes for patients recovering from MI. The list of important trials done in the recent past regarding the same is shown in Table 1 [15-25]. Key findings from systematic reviews, longitudinal studies, and RCTs highlight the benefits of HIIT in these populations.

**Table 1:** A literature review of various studies providing evidence regarding the efficacy of HIIT in clinical outcomes of cardiovascular disease patients. ACS: acute coronary syndrome; AIT: aerobic intensity training; BDI: Beck depression inventory; BDNF: brain-derived neurotrophic factor; BP: blood pressure; CABG: coronary artery bypass grafting; CAD: coronary artery disease; CHF: chronic heart failure; HIIT: high-intensity interval training; HRQoL: health-related quality of life; HR: heart rate; IHD: ischemic heart disease; LVEF: left ventricle ejection fraction; MCT: moderate continuous training; MI: myocardial infarction; MICT: moderate-intensity continuous training; MISS: moderate intensity steady state; RCT: randomized control trial; SF-36: short form 36; VO2: oxygen consumption

Author	Study Type	Study Population	Sample Size	HIIT Protocol	Control	Outcomes	Results	Conclusion
Wisløff et al. [15] 2007 Norway	RCT trial	Postinfarction heart failure patients	27 (mean age 75.5 ± 11.1 years)	HIIT: 95% max HR, 3x per week, for 12 weeks	MCT: 70% of max heart rate, 3x per week for total 12 weeks	VO₂ peak, LVEF, cardiac remodeling, endothelial function, quality of life	VO₂ peak increased more with HIIT (46%) than with MCT (14%, p < 0.001). Only the HIIT group significantly improved LVEF, and LV volume levels. Both groups improved quality of life scores.	Higher-intensity training is essential for reversing LV remodeling and improving overall cardiovascular health in post-infarction heart failure patients.
Moholdt et al. [16] 2009 Norway	RCT trial	Post CABG patients	59	AIT: 90% max HR, 5x per week, for 4 weeks	MCT: 70% max heart rate, 5x per week for 4 weeks	Vo₂ peak, quality of life	Both groups increased VO2 peak from baseline to 4 weeks, with the AIT group improving further by 6 months. Quality of life also improved in both groups.	Both AIT and MCT provide short-term benefits after CABG, but AIT leads to superior long-term improvements in VO₂ peak.
Conraads et al. [17] 2015 Belgium	Multicenter, prospective, RCT trial	CAD patients	200 (mean age 58.4 ± 9.1 years)	AIT: 95% peak HR, 3x per week, for a total 12 weeks	MCT: 70% peak HR, 3x per week, for total 12 weeks	Cardiovascular risk factors, safety, VO₂ peak, endothelial function, HRQoL	Peak VO₂ augmented in AIT (22.7%) and MCT (20.3%; p < 0.001). Flow-mediated dilation improved more in AIT (+34.1%) than in MCT (+7.14%; p < 0.001). Equal improvement of cardiovascular risk factors & quality of life.	AIT and MCT produced comparable improvements in peripheral endothelial function and exercise capacity in CAD patients, contradicting earlier trials with different outcomes.
Jaureguizar et al. [18] 2016 Spain	Randomized clinical trial	IHD patients	72	HIIT: 3x per week, for 8 weeks	MCT: for 8 weeks	Vo₂ peak, aerobic threshold, quality of life, 6-minute walk distance test, HRQoL	HIIT amplified VO₂ peak by 4.5 ± 4.7 vs. MCT's 2.5 ± 3.6 mL·kg·min (p < 0.05) and 6-minute walk by 49.6 ± 6.3 m, MCT by 29.6 ± 12.0 m (p < 0.05). HRQoL improved in both groups.	HIIT enhances quality of life and functional capacity in cardiac rehabilitation without increasing cardiovascular risk.
Nytrøen et al. [19] 2019 Norway	Multicenter, prospective, RCT trial	Heart transplant patients	81 (73% men)	HIIT: 85%–95% of peak effort, at 4×4-minute intervals, for 9 months	MICT: 60%–80% of peak effort for 9 months	VO₂ peak, anaerobic threshold, health-related quality of life, muscular strength, body composition	The HIIT group showed greater improvements in VO₂ peak, anaerobic threshold, and muscle capacity compared to the MCT group. HRQoL was similar, with no serious adverse events reported.	HIIT is safe and effective for improving exercise capacity in heart transplant recipients (de novo patients) compared to the MICT group.
Villafaina et al. [20] 2020 Spain	RCT trial	ACS patients	21 (HIIT and MICT groups)	HIIT: 90% HR with 15 seconds recovery, 10 sets of 15 seconds each, 2x per week, total 12 weeks	MICT for 40 minutes at 70%-75% HR, performed 2x per week for 12 weeks	Physical fitness, Body composition, HR variability, HRQoL	Both HIIT and MICT improved agility and mental HRQoL. HIIT showed greater gains in flexibility and handgrip strength (p < 0.05), with no substantial effects on cardiorespiratory fitness or HR variability.	HIIT is an effective and safe rehabilitation alternate for ACS patients, offering flexibility and strength benefits compared to MICT.
Papathanasiou et al. [21] 2020 Bulgaria	Single-blind, prospective RCT trial	CHF patients	120 (mean age 63.73 ± 6.68 years)	m-Ullevaal protocol: 3 sittings per week for a total of 12 weeks	MICT: 3 sittings per week for a total 12 weeks	6-minute walk test VO₂ peak, LVEF, perceived exertion scale, HRQoL	Both interventions significantly improved functional exercise capacity, LVEF, perceived exertion, and HRQoL (p < 0.001), with the m-Ullevaal protocol showing greater gains than MICT (p < 0.001).	The m-Ullevaal protocol is more effective than MICT for improving the quality of life and exercise capacity in CHF patients, and physicians can effectively implement it in cardiac rehabilitation.
Taylor et al. [22] 2020 Australia	Randomized clinical trial	Angiographically proven CAD patients	96	HIIT: 85%–95% of peak effort, at 4×4-minute intervals for 4 weeks, then home-based for 48 weeks	40-min MICT, 3 sessions/week for 4 weeks, then home-based for 48 weeks	VO₂ peak, feasibility, cardiovascular risk factors, safety, adherence, HRQoL	HIIT improved VO₂ peak by 10% after 4 weeks compared to 4% for MICT (P = .02). At 12 months, improvements were similar: HIIT at 10% and MICT at 7% (P = 0.30). Both groups demonstrated good feasibility and a little withdrawal rates.	HIIT is safe and effective, showing greater short-term VO₂ peak improvements than MICT, with similar long-term effects and adherence rates.
Yakut et al. [23] 2021 Turkey	RCT trial	MI patients	21	HIIT: 85–95% of HR reserve, 2x per week, total 12 weeks	MICT: 70–75% HR reserve, 2x per week, total 12 weeks	6-minute walk test, HR, BP, pulmonary function, respiratory muscle strength, HRQoL	Both HIIT and MICT improved resting blood pressure, HR, and functional capacity, However, HIIT was better than MICT in enhancing lower limb muscle strength and pulmonary functions (p < 0.05)	Both HIIT as well as MICT can be effectively useful at home for post-MI patients, significantly improving functional capacity and health outcomes.
Reed et al. [24] 2022 Canada	RCT trial	Post-revascularization CAD patients	135	HIIT: 4 × 4 min at 85%-95% peak HR, 2x per week, total 12 weeks	MICT: resting HR + 20-40 bpm, twice weekly for total 12 weeks	6 min walk test, depression (BDI-II), BDNF, quality of life (SF-36, HRQoL)	BDI-II scores improved across all groups, while BDNF levels remained unchanged. Significant enhancements were noted in SF-36 and HRQoL	All exercise programs were safe and well attended, with HIIT showing superior improvements in functional capacity, predicting future cardiovascular events.
McGregor et al. [25] 2023 UK	RCT trial	CAD patients	382 (HIIT: 187, MISS: 195)	HIIT: 10 × 1 min intervals at >85% peak HR, 2x per week, for eight weeks	MISS: 20-40 min at 60-80% peak HR, 2x per week, for eight weeks	VO₂ peak, cardiac structure and function, adverse events, HRQoL	VO₂ peak enhanced more with HIIT (2.37 mL/kg/min) than with MICT (1.32 mL/kg/min; P = 0.002). Only a single HIIT-related major adverse event was reported.	HIIT in low volume is safe and more effective than MISS for enhancing cardiorespiratory fitness in CAD patients, making it a valuable option for cardiac rehabilitation programs.

A systematic review by Wang et al. concluded that HIIT significantly increases exercise and endurance capacity as well as quality of life in post-MI patients [26]. This review included data from multiple RCTs, reinforcing the strength of HIIT as an effective rehabilitation strategy. It found that HIIT led to better enhancements in exercise capacity when compared to MICT, with an associated increase of 1.13 mL/kg/min in VO2 peak. Long-term studies have shown that patients who engage in HIIT post-MI experience lower rates of hospital readmission and improved survival rates. A cohort study by Jeong et al. found that patients participating in HIIT had a 30% reduction in all-cause mortality compared to those who did not engage in structured exercise programs [27]. A systemic meta-analysis of Weberg et al. found that participants engaging in HIIT experienced significant enhancements in health-related quality-of-life (HRQoL) measures, together with physical functioning and emotional well-being [28]. Heart disease health-related quality of life questionnaire (MacNew) showed substantial improvement from baseline (T1) to rehabilitation (T2) (P < 0.05). Also, almost all domains for the emotional role showed moderate to large effect size following HIIT interventions.

Continuously growing evidence supports the efficacy of HIIT in stroke rehabilitation. The list of important trials done regarding the same in the recent past is shown in Table 2 [29-37]. Key findings from randomized controlled trials and studies on cognitive function highlight the benefits of HIIT for stroke survivors. A randomized controlled trial by Oberlin et al. demonstrated that stroke survivors who participated in HIIT showed greater improvements in walking speed, balance, and overall fitness compared to those receiving standard rehabilitation [38]. They also found that exercise can complementary treatment for reducing depressive episodes and symptoms in patients after stroke. HIIT leads to significant enhancements in executive function and memory in stroke patients, potentially due to increased cerebral blood flow and neuroplasticity. Sakai et al. in their scoping review found that 24 HIIT treadmill sessions in blend with standard care are greater than standard care alone for improving balance, walking distance, and executive function [39]. They also consistently reported that HIIT is safe for post-MI and post-stroke patients, with statistically insignificant differences in the incidence of adverse events between HIIT and MICT groups.

**Table 2:** A literature review of various studies providing evidence regarding the efficacy of HIIT in clinical outcomes of cerebrovascular disease patients. AIH: acute intermittent hypoxia; AV O2: arteriovenous oxygen difference; BBS: Berg balance scale; BDNF: brain-derived neurotrophic factor; DGI: dynamic gait index; EQ-5D: EuroQol 5 Dimension; FIM: functional independence measure; HIGT: high-intensity gait training; HIIT: high-intensity interval training; HR: heart rate; MICT: moderate-intensity continuous training; NIHSS: National Institutes of Health Stroke Scale; RCT: randomized control trial; TMT-B: Trail Making Test-B; VO2: oxygen consumption

Author	Study Type	Study Population	Sample Size	HIIT Protocol	Control	Outcomes	Results	Conclusion
Boyne et al. [29] 2015 USA	Randomized crossover trial	Ambulatory persons with chronic stroke of more than 6 months.	19	30-second fast walking on a treadmill at max tolerated speed with varying rest periods of 30s, 60s, and 120s.	None specified	V̇O2 peak, Exercise tolerance, HR, peak treadmill speed, total step count	The P30 group got the highest mean VO2 at 70.9% VO2peak and 1,619 steps, but lower exercise tolerance than P60 or P120; P60 showed balanced results.	Combining P30 and P60 in HIIT could optimize treadmill speed, aerobic intensity, and stepping frequency, potentially enhancing gait and aerobic capacity.
Nepveu et al. [30] 2017 Canada	RCT trial	Patients with chronic stroke	22	High-intensity interval training (15 minutes) after motor task practice	Non-exercise control	Motor skill retention, neuroplasticity measures (corticospinal excitability)	HIIT improved motor skill retention compared to controls, with modest neuroplastic changes observed in participants.	One HIIT session after motor practice improved skill retention and potentially accelerated recovery in stroke patients.
Parreiras et al. [31] 2019 Brazil	Double-blind randomized trial	Stroke patients with weakness of respiratory muscle (N=38)	38	Home-based, high-intensity home respiratory muscle training, 40 minutes daily for 8 weeks, progressed weekly	Similar design but a sham intervention.	Primary: Strength of Inspiratory muscle; Secondary: Strength of expiratory muscle, walking capacity, inspiratory endurance.	Increased inspiratory strength by 27 cmH2O and expiratory strength by 42 cmH2O, reduced dyspnea by 1.3 points post-training, but no differences in respiratory complications or walking capacity.	Home-based high-intensity respiratory muscle training effectively improves respiratory strength and reduces dyspnea in chronic stroke patients.
Tong et al. [32] 2019 China	Pilot trial (RCT)	Mild-moderate ischemic stroke within 24 hours.	300	Early Routine Mobilization: < 1.5 h/d (24-48 h); Early Intensive Mobilization: ≥3 h/d (24-48 h); Very Early Intensive Mobilization: ≥3 h/d (< 24 h)	Standard care according to guidelines	Modified Rankin Scale score (0-2) at 3 months follow-up	The Early Intensive Mobilization group showed 53.5% favorable outcomes compared to 37.8% in the Very Early Intensive Mobilization group, indicating better rehabilitation effectiveness at 24-48 hours post-stroke.	High-intensity physical rehabilitation should commence 48 hours after a stroke for potential benefits. Very Early Intensive Mobilization has no favorable outcomes at 3 months.
Soh et al. [33] 2020 South Korea	Single-blinded RCT	Patients after minor stroke (NIHSS ≤3)	36	Lateral push-off skater exercise, 30 min, 3x per week for 12 weeks	Conventional treadmill training	EQ-5D, VO2peak, minute ventilation, Berg Balance Scale (BBS), Dynamic Gait Index (DGI),	Significant improvements in EQ-5D, VO2peak, oxygen pulse, minute ventilation, DGI, and BBS in the study group (P<.05)	Skater exercise significantly improves cardiorespiratory fitness, health-related quality of life, and balance, and is recommended for minor stroke patients.
Hsu et al. [34] 2021 Taiwan	RCT trial	Stroke patients (age ~55, stroke duration >24 months)	23	High-intensity interval training (HIIT) at 80% followed by 40% of VO2peak in alternate cycle	MICT at 60% of VO2peak for 36 sessions of 30 minutes each	VO2peak, CO, AV O2difference, cerebral oxygenation (Δ[O2Hb], Δ[HHb], Δ[THb]), BDNF levels, neuron morphology	HIIT significantly increased VO2peak by 20.7% (compared to 9.8% for MICT), cardiac output, Δ[HHb], Δ[THb], and serum BDNF levels, while also enhancing neurite percentage.	HIIT is more effective than MICT in improving aerobic capacity, cerebral oxygen utilization, serum BDNF levels, and neuronal activities in stroke patients.
Tor Ivar Gjellesvik et al. [14] 2021 Norway	Multicenter RCT	Adult stroke survivors (3 months to 5 years post-stroke)	70	4×4 minutes of HIIT by treadmill at 85%-95% of peak HR combined with standard care	Standard care alone	Physical, mental, and cognitive functioning are assessed using various tests (FIM and Stroke Impact Scale).	Significant enhancements in BBS, 6-minute walk test, and executive function (TMT-B) immediately after HIIT and after 12 months	HIIT combined with standard care significantly improves physical and cognitive function post-stroke, with lasting effects on executive function.
Krawcyk et al. [35] 2023 Denmark	Post-intervention follow-up RCT	Patients having a lacunar stroke	71	Home-based HIIT for three months with weekly motivational calls	Usual care	Cardiorespiratory fitness, physical activity, cognition, and mental well-being and recurrent stroke.	No change in cardiorespiratory fitness; increased vigorous-intensity activity post-intervention; similar recurrent stroke rates (n=3) in both groups.	With early HIIT, long-term cardiorespiratory fitness did not show significant improvement but increased post-stroke vigorous activity. Motivation is crucial for long-term physical activity.
Moncion et al. [36] 2024 Canada	Multi-site RCT	Individuals ≥6 months post-stroke	82	10×1-minute intervals at 80%-100% HRR interspersed with 1 minute at 30% HRR	MICT	Cardiovascular risk factors, V̇O2peak, mobility parameters (10 m gait speed, 6-minute walk test)	After 12 weeks, the HIIT group showed greater V̇O2peak gains (3.52 mL/kg·min) than MICT group (1.71 mL/kg·min), with no adverse events.	Short-interval HIIT may effectively improve V̇O2peak compared to MICT after 12 weeks, offering better cardiovascular fitness.
Hornby et al. [37] 2024 USA	Randomized crossover trial, Phase II	Chronic stroke patients (>6 months post-stroke)	35	AIH combined with HIGT for up to 15 sessions; AIH involved cycles of hypoxia (8%-9% O2) and normoxia (21% O2) before HIGT	Normoxia before HIIT	Peak treadmill speed, fastest speed, self-selected speed, 6-minute walk test,	AIH + HIGT: greater improvements in self-selected (0.14 m/s), fastest (0.16 m/s), peak treadmill speed (0.21 m/s), & spatiotemporal symmetry compared to normoxia + HIGT (P<0.01).	The combination of AIH and HIT significantly enhanced locomotor function compared to normoxia plus HIT, highlighting its potential to improve rehabilitation outcomes for stroke patients.

Program structure and protocols for HIIT

Implementing HIIT in cardiac and stroke rehabilitation programs requires careful planning and consideration. Below are detailed strategies for effective implementation, focusing on individualized protocols, integration with standard care, and training for healthcare providers.

Patient Selection Criteria for HIIT in Cardiac and Stroke Rehabilitation

When selecting patients for HIIT in cardiac rehabilitation, it is crucial to assess their cardiac risk profile. Patients with low to moderate cardiac risk are generally appropriate candidates. A systematic review by Way et al. found that HIIT can be relatively safe for patients with CVD, including CAD and heart failure when applied within structured rehabilitation settings [40]. Assessing functional capacity is essential for determining suitability for HIIT. The 6MWT is commonly used to assess baseline functional capacity; patients should be able to walk at least 150 meters during this test to engage in HIIT safely. Additionally, the Borg Rating of Perceived Exertion (RPE) scale (6 to 20) can help gauge perceived exertion during initial assessments. A scale of more than 15 is a relative contraindication for HIIT [40]. Baseline use of cardiac biomarkers such as troponin value or N-terminal pro-B-type natriuretic peptide can also be used to assess the baseline and after-exercise cardiac stress. Kumar et al. in their study have shown the prognostic importance of high-sensitivity troponin values in cardiovascular outcomes [41]. Patients must demonstrate a minimum level of functional capacity and stability before starting HIIT to ensure safety. Those with significant arrhythmias, unstable angina, or recent heart failure may require a more cautious approach and should be evaluated by a cardiologist before participating in HIIT.

For stroke survivors, the selection criteria should consider the severity and type of stroke. Patients who have experienced a stroke for the first time (infarct or bleed) and can walk without any support are generally suitable candidates for HIIT. Those with severe impairments or who require significant assistance may not be appropriate for high-intensity training. Ito et al. in their study used assessment scores such as the BBS score and Functional Ambulation Category (FAC) to evaluate a patient's balance and mobility [42]. Patients should have a minimum score on these assessments to ensure they can safely participate in HIIT. A BBS score (≥49/56) and FAC score (of ≥4/6) indicate sufficient balance to engage in higher-intensity activities. Cognitive assessment is also important, as some stroke patients may experience cognitive deficits that could affect their ability to follow instructions during HIIT. Ensuring that patients can comprehend and safely execute the training regimen is vital for their safety and success.

HIIT Protocol

**Figure 2:**
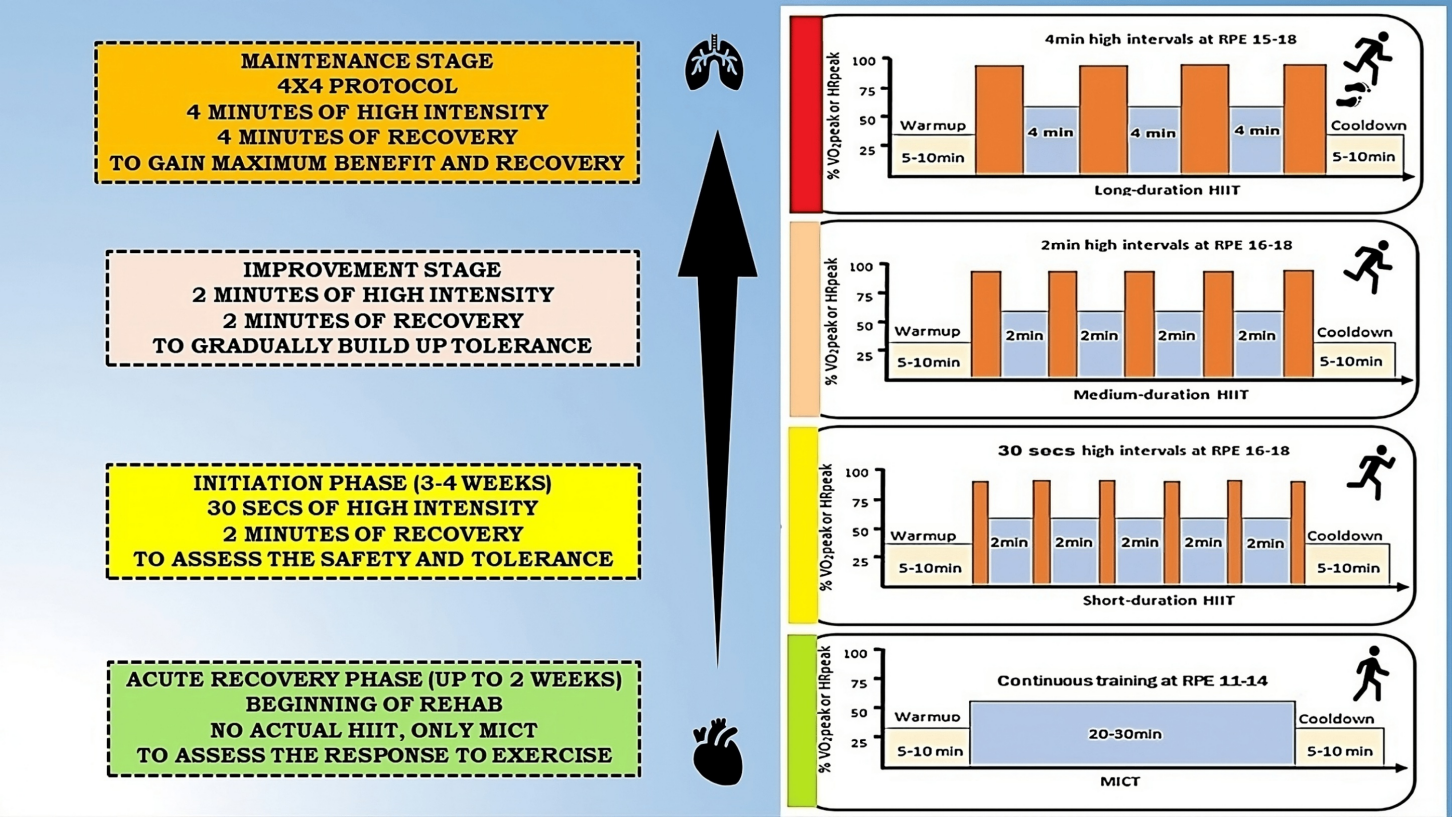
High-intensity interval training (HIIT) progression protocol with an overall timeline used within cardiovascular rehabilitation program. HIIT: high-intensity interval training; HRpeak: peak heart rate; MICT: moderate-intensity continuous training; RPE: rating of perceived exertion on 6-20 Borg scale; VO2peak: peak oxygen consumption Image Credit: Authors

Gayda et al. in their detailed review have documented wide varieties of interval training models for the general population as well as cardiovascular disease patients [43]. Some of the commonly used protocols are shown in Table 3. For the usual case of HIIT protocol, individuals are aimed for 2 to 3 sessions per week, with each session lasting about 20 to 30 minutes (Figure 2). This duration is divided into a warm-up period, exercise interval, and cool-down period.

**Table 3:** Common high-intensity and interval exercise training protocols used in cardiovascular rehabilitation. CPET: cardiopulmonary exercise testing; HIIT: high-intensity interval training; HR: heart rate; MI: myocardial infarction

Type of HIIT Protocol	Description	Intensity Structure	Target Population	Example Exercises
Short-interval HIIT	Brief bursts of high-intensity exercise (15-60 seconds) followed by short recovery (15-120 seconds).	85%-100% peak HR	Relatively young and stable post-MI & stroke patients	Treadmill Sprints, cycling sprints
Medium-interval HIIT	1-2 minutes of high-intensity exercise with 1-4 minutes of low-intensity recovery.	70%-90% peak HR	Patients recovering from MI or stroke	Jogging followed by walking
Long-interval HIIT	Longer high-intensity efforts (4 minutes) with extended recovery periods (3 minutes).	85%-95% peak HR	Older patients, post-MI or stroke	Rowing or cycling at high intensity
Low-Volume HIIT	Shorter sessions focusing on fewer intervals but maintaining high intensity for safety and adherence.	70%-85% peak HR	Older patients recovering from MI or stroke or with multiple comorbidities	Cycling at high intensity for 1 minute, rest for 2 minutes
Fartlek training	Blends continuous running with random bursts of speed.	Variable	Runners recovering from cardiac and stroke events	Sprinting between trees during a run
Tabata protocol	A specific form of HIIT lasting 4 minutes, consisting of 20 seconds of ultra-intense exercise followed by 10 seconds of rest, repeated for 8 rounds.	Near-maximal to supra-maximal effort, > 110% of peak HR and VO2 max	The general population, including those with CVD	Bodyweight exercises like burpees or squats
EMOM (Every Minute on the Minute)	Perform a set number of repetitions at the start of each minute, resting for the remainder.	Variable	Patients with advanced levels of exercise training	Push-ups or kettlebell swings
Supervised HIIT in rehabilitation	A structured program involving multiple sessions per week focusing on safety and gradual intensity increase.	Personalized based on CPET results	Patients in rehabilitation post-MI & post-stroke	Treadmill intervals or recumbent cycling

Warm-up: The warm-up is essential for preparing one’s body for the exercise. It progressively increases heart rate and blood flow to the muscles recruiting more sarcomere and muscle fiber for the active contraction phase. Activities should include light aerobic exercises and dynamic stretches focusing on major muscle groups. An example routine could consist of five minutes of brisk walking or cycling followed by five minutes of dynamic stretching, including movements like torso twists and ankle circles.

Intensity and duration: The intensity of HIIT is often measured by percentage utilization of VO2 max, HR max, run velocity max, peak power output in watts, or Borg RPE. HIIT should be performed at 80-90% of the maximum heart rate reserve. It can be applied to several types of exercise such as cycling, running, swimming, rowing, and whole-body exercise. The duration of high-intensity intervals can vary based on the patient's fitness level. The recommended approach for a cardiovascular disease patient is divided into 3 stages. The first stage is initiation (0-4 weeks of acute event) and in this stage, only continuous aerobic exercise training (CAET) is allowed (up to 75% of VO2 max) with no actual HIIT sessions. The second stage is improvement (4-12 weeks of the acute event) in which HIIT starts with 30 seconds of high intensity, followed by 30 seconds of recovery. The third stage is maintenance (>12 weeks of the acute event) in which patients should aim for intervals of one to four minutes of high-intensity work-out, followed by equal or slightly longer recovery periods.

Recovery: The recovery period involves lower-intensity activities, allowing the heart rate to decrease adequately before the next interval. Recovery can be of various intervals - from 15 seconds up to 2-3 minutes in duration depending upon the amount of work performed during the high-intensity phase. Recovery prevents the overcollection of lactic acid at a toxic level due to anaerobic ATP generation by partially washing it away for skeletal muscle. It also provides some time for the selected group of sarcomeres to relax and prevent physical injury to muscles. Encouraging active recovery, such as slow walking or light cycling, helps maintain blood flow.

Cool-down: The cool-down phase involves keeping the body moving at a lesser intensity after exercise. Expending additional time on this phase has numerous benefits, such as preventing blood pooling in leg veins and promoting increased blood circulation. It gradually returns the blood pressure, heart rate, and temperature of the body to normal resting levels. By reducing lactic acid levels in the muscles used during exercise, the cool-down phase also helps decrease muscle tightness and cramping and delayed onset muscle soreness. This ultimately benefits maintaining good muscle length and flexibility. Activities should include slow walking or cycling at a low intensity for about five minutes, followed by static stretching focusing on major muscle groups used during exercise (e.g., hamstrings, quadriceps, shoulders). Other examples are Jefferson curls, deep squat holds, deep lunge hip flexor stretches, Cossack squat stretches, cobra pose, downward dog, stretch, etc.

Safety Considerations for HIIT in Cardiac Rehabilitation

Given the higher intensity workout in HIIT, it is important to assess its safety level in people with elevated cardiometabolic risk. Guiraud et al. in their study among patients with CAD and chronic heart failure found no significant ECG changes, such as ST-segment depression or elevation, blood pressure drops, and syncope during interval or recovery periods of HIIT [44]. However, there are some absolute contraindications for HIIT such as unstable angina, recent myocardial infarction within one-month, single chamber pacemaker, severe symptomatic aortic stenosis, uncontrolled hypertension, symptomatic stroke within 1 month with residual significant disability, acute pulmonary embolism and other acute non-cardiac or non-neurological disorder. Comorbidities (e.g., diabetes, hypertension) may affect exercise capacity and set specific rehabilitation goals (e.g., improving endurance or strength training). The presence of anemia reduces the body's ability to carry oxygen, leading to lower endurance levels during HIIT. In a study by Qureshi et al., it was found that anemia, absolute iron deficiency, and functional iron deficiency in the body are associated with poor cardiovascular outcomes [45]. Therefore, special attention should be paid to correcting anemia. By following the structured approach to HIIT, healthcare providers can ensure that cardiac rehabilitation patients engage in effective and safe exercise sessions that promote recovery and improve overall cardiovascular health.

HIIT sessions should be supervised by qualified healthcare professionals, such as exercise physiologists or physical therapists. This ensures proper technique is monitored and immediate assistance can be provided if needed as it allows for real-time adjustments based on patient responses. Continuous monitoring of blood pressure, heart rate, and oxygen saturation is essential during HIIT sessions. Healthcare providers should observe patients for signs of fatigue, abnormal heart rhythms, and fluctuating blood pressure. It is crucial to have emergency protocols in place, including staff trained in CPR and the use of an automated external defibrillator. Patients should be encouraged to communicate any discomfort immediately. Increased awareness can enhance patient confidence and safety during high-intensity training.

Integration of HIIT with standard care in cardiac and stoke rehabilitation

By integrating HIIT with standard care practices, healthcare providers can create a holistic rehabilitation program that addresses physical, psychological, and nutritional needs, ultimately leading to better health outcomes for patients recovering from cardiovascular events. By focusing on strategies like motivation through gamification, incorporating patient feedback, and educating participants on the benefits of HIIT, healthcare providers can improve adherence and acceptance of HIIT programs among patients recovering from cardiovascular events or strokes.

Holistic Approach and Collaborative Team Care

Integrating HIIT with standard care practices is essential for comprehensive cardiac rehabilitation. It is important to consider the following aspects for cardiovascular health: medication management, ensuring patients are on appropriate medications, dietary counseling to provide nutritional guidance, and psychosocial support to address mental health needs. Proper medication management can enhance the effectiveness of exercise interventions and reduce cardiovascular risk, a balanced diet complements the benefits of HIIT, and addressing mental health needs significantly impacts adherence to rehabilitation programs and overall recovery outcomes. Encouraging collaboration among healthcare providers, including cardiologists, dietitians, physical therapists, and psychologists, is key to creating a comprehensive and individual plan of action for each patient. This multidisciplinary approach safeguards the various aspects of a patient’s health, leading to improved outcomes. Research indicates that collaborative care models enhance patient satisfaction and adherence to treatment plans.

Patient Education, Counseling, and Feedback

Educating patients about the benefits of HIIT is critical for promoting engagement in their rehabilitation process. Patients should be informed about how HIIT can improve quality of life, and cardiovascular fitness while also reducing the risk of future cardiovascular events. Addressing safety concerns and providing reassurance regarding the monitoring processes in place during HIIT sessions further enhances patient confidence and acceptance of the program. Informed patients are more likely to adhere to exercise regimens and report positive experiences during rehabilitation. Participants should be asked via feedback questionnaire, regarding their experience and difficulty level of HIIT using a 10-point Likert scale. This feedback can help healthcare providers adjust programs based on patient preferences and reported enjoyment levels, ensuring that the training remains relevant and appealing. Qualitative studies have shown that patient satisfaction is closely linked to adherence; those who feel their needs are being met are more likely to continue participating in the program.

Training for Healthcare Providers in HIIT Implementation

Healthcare providers are required to continuously enhance their ability to implement HIIT effectively within cardiovascular rehabilitation programs, to improve patient outcomes and adherence to exercise regimens. Clague-Baker et al. recently conducted a study to explore cardiovascular rehabilitation teams' knowledge, attitudes, and belief systems, as well as wider topics such as exercise, food, healthy lifestyles, addiction, and behavior changes for individuals in the sub-acute phase of recovery [46]. This study involved 12 focus groups with 57 health professionals from acute and community national health service trusts. Positive impacts of increased confidence were noted for the rehabilitation team and stroke survivors after the intervention, but some negatives and barriers were also recognized. There was a lack of knowledge among cardiac rehabilitation teams regarding stroke survivors, as well as among stroke teams regarding cardiac rehabilitation, exercise, and healthy lifestyles. It is important to address the confidence and knowledge gaps among cardiac rehabilitation and stroke teams to ensure better support for stroke survivors during their recovery.

To effectively implement HIIT in rehabilitation, it is essential to provide training workshops for healthcare providers. These workshops should focus on the principles of HIIT, safety considerations, and exercise prescriptions tailored specifically to cardiac patients. Practical sessions with hands-on training where providers can practice supervising HIIT sessions are crucial for building confidence and competence in delivering these programs. Encouraging ongoing education through seminars or online courses is vital for keeping healthcare providers updated on the latest research and best practices in cardiac rehabilitation. Continuous professional development ensures that providers are aware of new findings related to HIIT, including its efficacy, safety, and implementation strategies. Developing resources such as guidelines or checklists for healthcare providers can serve as valuable references when implementing HIIT in their practice. These resources should include information on exercise protocols, safety monitoring, patient selection criteria, and strategies for enhancing patient adherence. Providing clear, accessible guidelines helps standardize care and ensures that all team members are aligned in their approach to HIIT, which has been shown to improve compliance and safety in clinical settings.

Challenges of HIIT

HIIT offers significant benefits for patients recovering from MI and stroke, but its implementation comes with several challenges that must be managed assertively to ensure safety and effectiveness. One primary concern for MI patients is cardiac risk. Individuals with prior MI often have compromised cardiac function, making them particularly susceptible to variations in heart rate and blood pressure during high-intensity exercise. For stroke patients, neurological impairments present additional challenges. Therefore, exercise sessions should be supervised by qualified healthcare professionals to mitigate risks and ensure patient safety. The need for individualized protocols is another challenge. The intensity and duration of HIIT must be personalized to each patient's specific capacity, considering their cardiac status and overall fitness level. Determining optimal training parameters is an ongoing challenge in stroke rehabilitation. The varieties of HIIT protocols implemented in the various literature studies need attention. Most of the HIIT studies done clinically are short-term and performed in a controlled laboratory setting. The efficacy of longer-duration HIIT in a home-based, real-world scenario along with its acceptability requires more research before it can be accepted as a complementary therapy for cardiovascular patients and those with high cardiometabolic risk. Finding the right balance between intensity and recovery is crucial for maximizing benefits while minimizing risks. Patient adherence also poses a significant challenge. Educating patients about the benefits of HIIT, as well as the safety measures in place, is vital for improving participation rates and encouraging consistent engagement in the program.

## Conclusions

HIIT represents a promising method for the rehabilitation of myocardial infarction and stroke patients. Its ability to improve cardiovascular fitness, enhance metabolic health, and promote functional recovery makes it a valuable addition to traditional rehabilitation strategies. As research evolves, HIIT may play an increasingly central role in managing and rehabilitating these high-risk patient populations. Integrating HIIT into standard care protocols could significantly improve patient’s quality of life and outcome, ultimately reducing the burden of cardiovascular diseases. It is crucial to investigate the sustainability of HIIT benefits over extended periods and its impact on long-term cardiovascular health. More extensive trials comparing HIIT with other rehabilitation modalities in diverse patient populations, including those with different comorbidities and varying levels of functional capacity, are necessary. By addressing these areas, future research can solidify the role of HIIT in cardiac rehabilitation and provide insights into optimizing its application for various patient populations.
